# Days at home alive after major surgery in patients with and without diabetes: an observational cohort study

**DOI:** 10.1186/s13741-023-00357-5

**Published:** 2024-01-22

**Authors:** Amanda Habermann, Matilda Widaeus, Navid Soltani, Paul S. Myles, Linn Hallqvist, Max Bell

**Affiliations:** 1 Department of Anaesthesia and Intensive Care Medicine, Karolinska University Hospital, Stockholm, Sweden; 2 Department of Physiology and Pharmacology, Karolinska Institute, Stockholm, Sweden; 3 Department of Anaesthesiology and Perioperative Medicine, Alfred Hospital, Melbourne, VIC Australia

## Abstract

**Objective:**

We hypothesized that days at home alive up to 30 days after surgery (DAH30), a novel patient-centered outcome metric, as well as long-term mortality, would be impaired in patients with type 1 or 2 diabetes mellitus (DM) undergoing major surgery.

**Methods:**

This cohort study investigated patients > 18 years with and without DM presenting for major non-cardiovascular, non-ambulatory surgical procedures at 23 hospitals in Sweden between 2007 and 2014. We identified 290,306 patients. Data were matched with various quality registers. The primary outcome was the composite score, DAH30. The secondary outcome was mortality from 31 to 365 days. Using multivariable logistic regression, significant independent risk factors influencing the primary and secondary outcomes were identified, and their adjusted odds ratios were calculated.

**Results:**

Patients with DM type 1 and 2 had significantly lower DAH30 as compared to non-diabetics. Patients with DM were older, had higher co-morbid burden, and needed more emergency surgery. After adjustment for illness severity and age, the odds of having a DAH30 less than 15, indicating death and/or complications, were significantly increased for both type 1 and type 2 diabetes. In the year after surgery, DM patients had a higher mortality as compared to those without diabetes.

**Conclusions:**

The results of this large cohort study are likely broadly generalizable. To optimize patient and societal outcomes, specific perioperative care pathways for patients with diabetes should be evaluated.

**Supplementary Information:**

The online version contains supplementary material available at 10.1186/s13741-023-00357-5.

## Introduction

Diabetes mellitus (DM) is a global healthcare burden, with an estimated prevalence of 7.2–11.4% (Federation 2022), and is expected to rise in years to come (Andersson et al. 2015). DM has many subtypes but is largely divided into two categories: type 1 DM (absolute insulin deficiency) and type 2 (insulin resistance and relative insulin deficiency) (American 2007); type 2 diabetes accounts for 90% of cases (Tripathi and Srivastava 2006). Diabetic patients are more likely to require surgery compared to those without—the prevalence of diabetes in surgical patients is estimated to be 10–15% (Clement et al. 2004; Hoffman et al. 2014; Smiley and Umpierrez 2006). Perioperative hyperglycemia is associated with increased 30-day mortality (Frisch et al. 2010).

There are multiple sequelae of poor glycemic control with a risk of both micro- and macrovascular complications (American 2007; Emerging Risk Factors et al. 2010). Increased risk of perioperative infection among diabetic patients is presumed to be secondary to immune response modulation (Nicolini et al. 2018). Postoperative complications, in particular, stemming from infections, can result in prolonged length of stay (LOS), higher re-admission, and worsened surgical outcomes (Holt et al. 2021). A review of 61 perioperative studies published in 2022 reports associations with mortality, complications, and infections (Drayton et al. 2022). A meta-analysis from the same year showed that diabetic patients had an odds ratio (OR) of 1.65 for any complication following non-cardiac surgery (Zhang et al. 2022). Specifically, the DM group had higher ORs for infections, wound healing disorders, hematomas, renal insufficiency, and myocardial infarction postoperatively (Zhang et al. 2022).

Days alive and at home up to 30 days after surgery (DAH30) is a novel composite endpoint. In contrast to LOS, DAH30 incorporates hospital readmissions and discharge to nursing facilities (as opposed to home) and has therefore been proposed as a more accurate proxy for surgical outcome (Bell et al. 2019). DAH30 was significantly lower among high-risk patients and in those suffering serious postoperative complications (Bell et al. 2019). Myles et al. make the case that DAH30 is a patient-centered outcome, since readmission is a major adverse event (Myles et al. 2017). Moreover, in a recent study of a mixed surgical population, DAH30 correlates with the cost of medical care postoperatively, where a low score correlates to higher hospital costs (Reilly et al. 2022).

The present study used Orbit, a large national perioperative database encompassing 23 hospitals in Sweden, to study DAH30 for patients with type 1 and type 2 diabetes. We hypothesized that DAH30 would be lower for DM patients overall compared to non-diabetic counterparts and that type 1 diabetics would have even worse outcomes. We further hypothesized that long-term mortality for DM patients would be increased.

## Methods

The study protocol (2014/1306–31/3) was approved by the Regional Ethics Committee of Stockholm, Sweden.

**Fig. 1:**
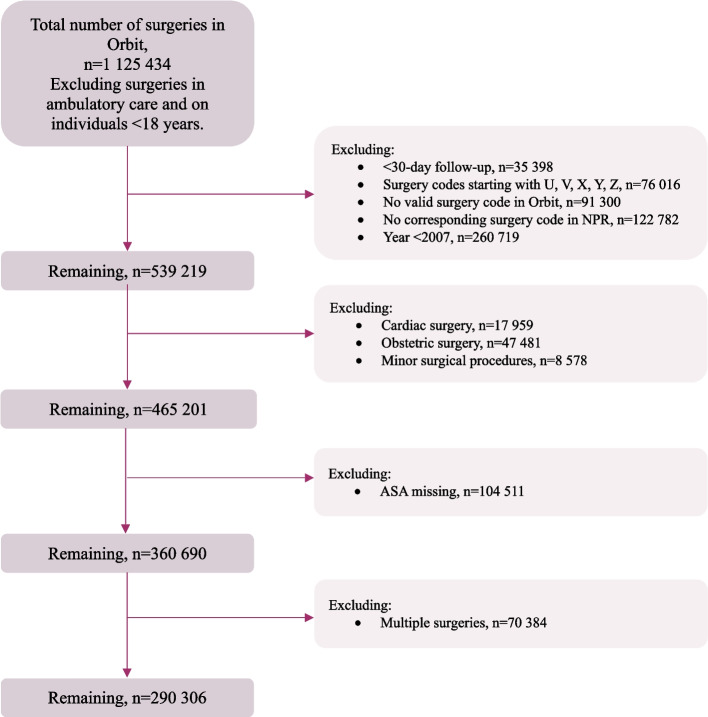
Flowchart. CONSORT flowchart of excluded subjects. *Abbreviations*: ASA, American Society of Anesthesiologists; *n*, number of subjects; NPR, National Patient Register

This observational multicenter cohort study uses the surgical planning software Orbit, with data from 23 Swedish hospitals at the university, county, and district levels. These sites cover roughly 40% of Swedish perioperative patients, and the hospitals are located throughout the nation. Patients aged ≥ 18 years, undergoing surgery from January 1, 2007, to December 31, 2014, were included. We excluded cardiac, obstetric, ambulatory, minor, or multiple surgeries and those lacking a valid surgery code in Orbit or a corresponding surgery code in the national patient register (NPR) (see data sources below). Furthermore, we excluded patients identified from hospitals with a high proportion of missing American Society of Anesthesiologists (ASA) physical status classification. Figure 1 provides a CONSORT flowchart.

### Data sources

#### Orbit

This is a surgical-perioperative planning software. It includes ASA, date, type, and duration of surgery and anesthesia, linked to the Swedish personal identification number (PIN) (Ludvigsson et al. 2009). Surgery cannot be performed without using Orbit, and computers with Orbit are present in all operating rooms. The system identified our cohort.

#### The Swedish national patient register, the Swedish prescribed drug register, and the cause of death register

Data extracted from Orbit were linked to the NPR using the unique Swedish PIN assigned at birth or immigration (Ludvigsson et al. 2009). The NPR provides information of all hospital admissions since 1987 and outpatient care since 2001 (Ludvigsson et al. 2011). In inpatient care, the visits missing a main diagnosis was 0.8% both in 2015 and 2021 (Socialstyrelsen 2018). The Swedish prescribed drug register contains information about all dispensed and collected drug prescriptions in Sweden since July 2005 (Welfare NBoHa 2023). Medications are classified according to the anatomical therapeutic code, ATC, and classification system (Socialstyrelsen 2018). In the Swedish Cause of Death Register, virtually all (> 99%) deaths are registered since 1952 based on the current ICD classification (Healthcare NBo 2023; Brooke et al. 2017).

After merging Orbit data with the abovementioned registers, we could extract patient characteristics and medical history, including age, sex, ASA class, preoperative diagnoses, and collected drug prescriptions. Data was retrieved from the NPR and the Swedish prescribed drug register for 5 years prior to index surgery. Data from the Swedish cause of death register was collected for 1 year after surgery. Perioperative characteristics included date, type, and duration of surgery and were extracted from Orbit. Surgical procedures were clustered according to the Nordic Medico-Statistical Committee classification (NOMESCO-NCSP) (NOMESCO 2009).

#### ICD coding and exclusions

Cardiovascular disease was classified as ICD-10, I10–I15, I20–I22, and I24–25. Peripheral artery disease was classified as ICD-10 I70–79. Cardiac disease was classified as ICD-10 I5–I8, I26–I28, I34–I37, I42, and I50. Arrhythmic disease was classified as ICD-10 I44–I49. Renal disease was classified as ICD-10 N11–N19 and N25–29. Cerebrovascular disease was classified as ICD-10 I60–I69. Pulmonary disease was classified as ICD-10 J10–18 and J44 (WHO 2019).

Inputs with < 30 days follow-up; surgery codes U, V, X, Y, and Z; invalid surgery codes; and an invalid surgery code in NPR were excluded. The U, X, Y, and Z codes were used for transluminal endoscopy, diagnostic interventions in surgery, organ harvesting, and addition codes, respectively, and were not studied in this project (Socialstyrelsen 2002). Surgery codes starting with V was—at the time of data collection—used for standardized care flow for cancer diagnostics (Socialstyrelsen 2018).

Cardiac surgery, obstetric surgery, and minor surgical procedures were excluded. Cardiac surgery was excluded because many of the studies on postoperative diabetic mortality have been done on cardiac surgery (Krolikowska et al. 2009). Obstetric surgery was excluded, due to the risk of diabetes gravidarum being misinterpreted under the same spectrum as diabetes mellitus. Minor surgical procedures were excluded so the study would only include major surgery.

#### Definitions of diabetes

Patients with DM were identified first based on ICD-10 codes for DM in NPR. DM 1 had an International Classification of Disease, Tenth Edition, ICD-10 code E10. DM 2 had ICD-10 code E11. DM unspecified had ICD-10 code E12.

Patients classified as having both DM 1 and 2 were sub-grouped into DM 2 if they had both diagnoses and collected antidiabetics other than insulin (using the ATC codes from the Swedish prescribed drug register), up to 5 years prior to surgery. Patients classified as having both DM 1 and DM 2 were sub-grouped into DM 1 if having collected an insulin prescription (ATC code A10B) and no other antidiabetic prescription (ATC code A10A), within 5 years prior to surgery. This was necessary due to suspected misclassification in the dataset; 5509 of 23,473 (all diabetics) were labeled as having both DM 1 and DM 2.

#### Calculating DAH30

DAH30 was calculated as previously described in detail (Myles et al. 2017)—from the date of index surgery (day 0) using hospitalization and mortality data up to day 30. If a patient dies in a hospital or after discharge anytime within the first 30 days after surgery, the patient is assigned 0 DAH30. A patient discharged from the hospital on day 3 after surgery but soon readmitted for 7 days before their second hospital discharge, then the patient would be assigned 20 DAH30. We report both mean DAH30 for patients with DM (type 1 and 2) and without DM. We also dichotomize DAH30—up to or under 15—where the latter is the worse outcome.

#### Statistics

Data was analyzed with Rstudio v. 2022.12.0 + 353 (Team RC. R n.d.). Continuous variables were presented as means and standard deviations, and categorical data was presented as numbers and percentages. *P* values were calculated and regarded as significant if < 0.05. *P* values of categorical data were calculated using Pearson’s chi-squared test and continuous variables using unpaired two-sample *t* test.

DAH30 and mortality were studied in separate analyses using multivariable logistic regression. Variables included in the multivariable analysis were chosen based on univariable analysis for each factor. In the univariate logistic regression, variables with a 95% confidence interval, CI 95% including the null hypothesis (OR = 1) were excluded (none). A backward stepwise regression model was used, and variables that were statistically insignificant (*P* > 0.05) were excluded (none, no variables yielded insignificant *P*-values) until the stopping rule was satisfied.

Proxy for comorbidity was two separate analyses: ASA and ICD-10. This separation was done to handle collinearity.

#### Data and resource availability

The datasets generated during and analyzed in the current study are available from the corresponding author upon reasonable request.

## Results

Figure 1 is the flowchart, showing the exclusions, detailed in the “Methods” section.

Table 1 details the demographics of the whole cohort, among all patients with DM and in patients with type 1, type 2, and unspecified diabetes mellitus. A total of 290,306 patients remained for analysis after exclusions, and 23,743 or 8.18% had DM, with the vast majority classified as type 2.

**Table 1:** Baseline characteristics of surgical cohort and proportion of diabetes

*Characteristics*	*Whole cohort*	*Non-diabetics*	*DM, all*	*DM 1*	*DM 2*	*DM unspecified*
*Number of subjects, no., %*	290,306, 100.00%	266,563, 91.82%	23,743, 8.18%	4569, 1.57%	19,071, 6.57%	44, 0.02%
*Mean DAH30, days*	24.16	24.40	21.41	21.53	*21.37*	18.61
*31-day-1-year mortality, no., %*	14,191, 4.89%	11,867, 4.45%	2324, 9.79%	451, 9.87%	1862, 9.76%	6, 13.64%
*P value, mortality*	–	–	< 0.001*	< 0.001*	< 0.001*	< 0.001*
*ASA class 1, %*	*29.55%*	*32.07%*	*1.33%*	*2.04%*	*1.15%*	*2.27%*
*ASA class 2, %*	*43.71%*	*44.22%*	*37.97%*	*39.88%*	*37.56%*	*27.27%*
*ASA class 3, %*	*24.61%*	*21.86%*	*55.40%*	*52.90%*	*55.99%*	*54.55%*
*ASA class 4, %*	*2.13%*	*1.84%*	*5.30%*	*5.19%*	*5.30%*	*15.91%*
*Women, %*	*55.26%*	*56.16%*	*45.13%*	*45.72%*	*44.98%*	*54.55%*
*Acute surgery, %*	*31.09%*	*30.84%*	*33.91%*	*38.52%*	*32.80%*	*50.00%*
*Cancer surgery, %*	*21.02%*	*20.94%*	*21.93%*	*15.08%*	*23.57%*	*13.64%*
*Top 3 most common surgery types, %*	*Orthopedic, 31.65%; abdominal, 19.58%; urologic, 10.15%*	*Orthopedic, 31.30%; abdominal, 19.77%; urologic, 9.98%*	*Orthopedic, 35.58%; abdominal, 17.46%; urologic 12.05%*	*Orthopedic, 36.18%; abdominal, 15.25%; vascular 8.78%*	*Orthopedic, 35.45%; abdominal, 17.96%; urologic, 12.87%*	*Orthopedic, 40.91%; abdominal, 11.36%; urologic, 11.36%*
*Top 3 most common comorbidities, %*	*Cardiovascular, 23.87%; arrhythmic, 13.59%; cardiac, 6.66%*	*Cardiovascular, 19.93%; arrhythmic, 12.12%; lung, 5.78%*	Cardiovascular, 68.18%; arrhythmic, 30.02%; cardiac, 19.26%	*Cardiovascular, 59.68%; renal, 24.44%; arrhythmic, 24.03%*	*Cardiovascular, 70.16%; arrhythmic, 31.33%; cardiac, 19.67%*	*Cardiovascular, 75.00%; renal, 38.64%; arrhythmic or cardiac, 34.09% each*
*Age, mean* ± *SD*	*60.13* ± *18.87*	*59.31* ± *19.06*	69.37 ± 19.06	*61.82* ± *18.89*	*71.16* ± *19.03*	*68.61* ± *14.49*

The non-diabetic and diabetic populations

*Abbreviations*: *ASA* American Society of Anesthesiologists, *DM 1* Diabetes mellitus type 1, *DM 2* Diabetes mellitus type 2, *SD* Standard deviation

^*^Pearson’s chi-squared test

The mean DAH30 was 24.4 among non-diabetics and 21.4 in the DM group, without major differences between type 1 and 2 DM. Mortality, measured as death between 31 and 365 days, was significantly higher in the perioperative diabetic population. The diabetic population has a higher ASA class (with very few patients misclassified as ASA 1) and different comorbid profile. Patients with diabetes were more likely to have cardiovascular disease and a diagnosis of arrhythmia. Among diabetes type 1, renal diseases were common. Patients with diabetes were older, and male sex dominated.

**Fig. 2:**
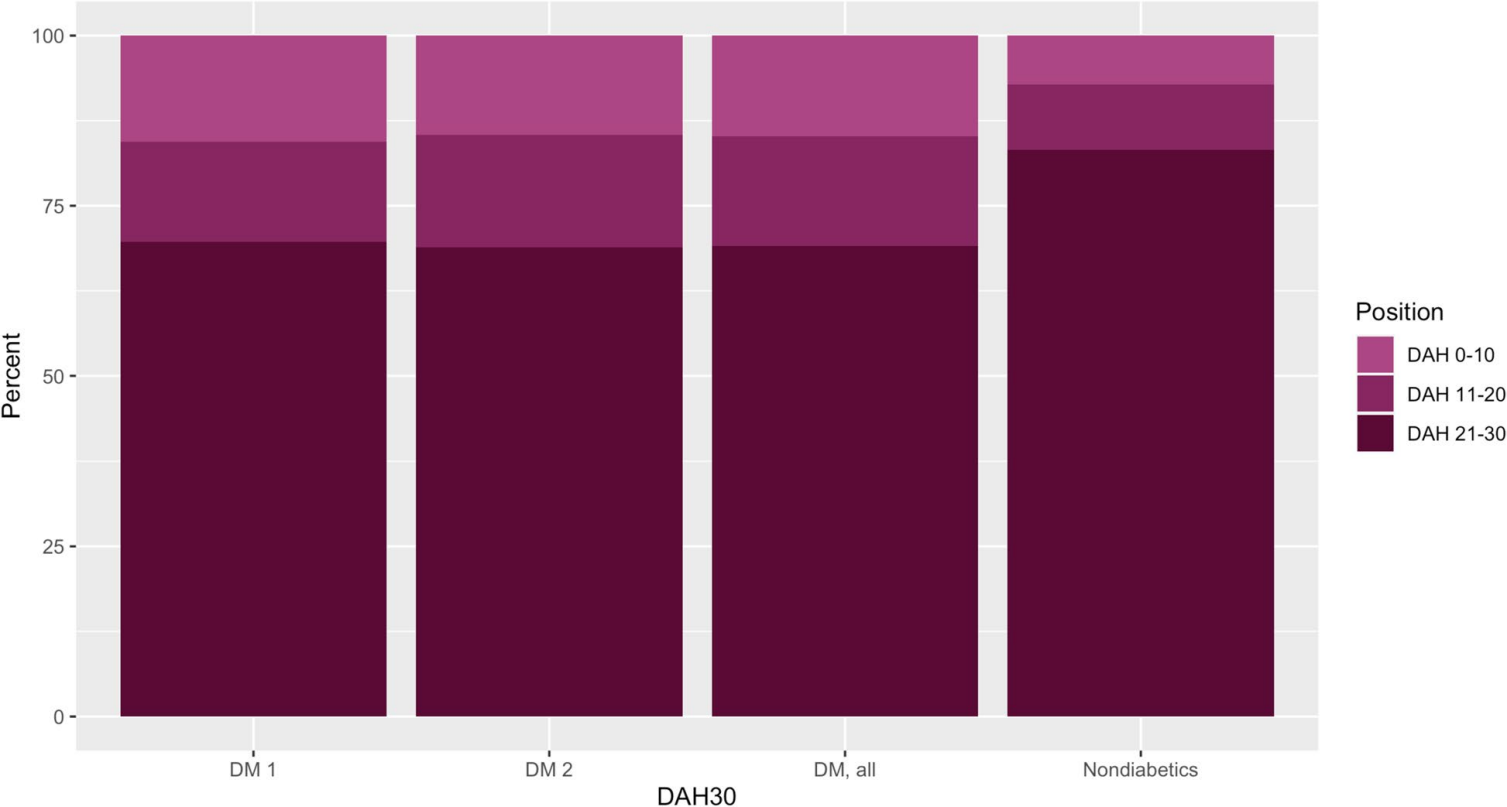
DAH30 strata for non-diabetics, diabetics in total, DM 1, and DM 2. DAH30 strata for diabetics. *Abbreviations*: DAH30, days at home and alive, DM 1, diabetes mellitus type 1; DM 2, diabetes mellitus type 2

Figure 2 illustrates the distribution of DAH30 among patients with and without DM, including the DM1 and DM2 subgroups. Days at home after surgery within 30 days are impacted by diabetes, indicating higher mortality, prolonged hospital stay, and/or readmissions within 30 days. Very low DAH30 (10 or lower) was more common in patients with diabetes.

Table 2 presents the univariate and multivariable analyses (ASA class used to adjust for comorbidity) where the outcome DAH30 was dichotomized up to/under 15. Several findings are worth highlighting. The risk of low DAH30 was significantly elevated among both type 1 and 2 DM, but more pronounced among diabetes type 1 after full adjustment. Higher ASA class was associated with low DAH30, with ASA 3 patients having a sevenfold increased risk for this adverse outcome in the adjusted model. Interestingly, DAH30 improved somewhat in the latter part of the study period. Acute surgery was associated with more than a doubled risk of DAH30 < 15. Cancer and neurosurgery were coupled with close to a threefold risk of low DAH30. In the crude model, vascular surgery was associated with a slightly elevated risk of DAH < 15 but not after adjustments for confounders. Ophthalmic, ENT, oral, and maxillofacial surgery all had very low risks of both low DAH30 and long-term mortality.

**Table 2:** Crude and adjusted odds ratios for DAH30 with ASA as a proxy for comorbidity

Characteristics	Crude OR, DAH30 < 15 ± 95% CI	Adjusted OR, DAH30 < 15 ± 95% CI
Non-diabetics	1.00 (reference)	1.00 (reference)
DM, type 1	2.17 (2.01–2.33)	1.42 (1.30–1.54)
DM, type 2	2.14 (2.06–2.23)	1.13 (1.09–1.19)
DM, unspecified	3.67 (1.85–6.85)	1.44 (0.66–3.01)
Age	1.04 (1.04–1.04)	1.01 (1.01–1.02)
Women	0.80 (0.78–0.82)	0.96 (0.94–0.99)
Men	1.00 (reference)	1.00 (reference)
**ASA class**		
ASA 1	1.00 (reference)	1.00 (reference)
ASA 2	3.66 (3.47–3.86)	2.67 (2.52–2.83)
ASA 3	15.20 (14.40–16.00)	7.32 (6.91–7.76)
ASA 4	68.70 (64.00–73.70)	25.60 (23.70–27.60)
Year of surgery		
2007–2010	1.00 (reference)	1.00 (reference)
2011–2014	0.91 (0.89–0.93)	0.75 (0.73–0.77)
Type of surgery		
Acute	3.25 (3.17–3.33)	2.71 (2.63–2.79)
Elective	1.00 (reference)	1.00 (reference)
Cancer	1.61 (1.57–1.65)	2.75 (2.65–2.85)
Neuro	3.84 (3.72–3.97)	2.90 (2.78–3.03)
Endocrine	0.09 (0.07–0.11)	0.17 (0.13–0.20)
Ophthalmic	0.12 (0.09–0.15)	0.14 (0.11–0.19)
Ear, nose, and throat	0.19 (0.16–0.22)	0.36 (0.31–0.42)
Oral and maxillofacial	0.19 (0.17–0.22)	0.35 (0.31–0.40)
Thoracic non-cardiac	3.00 (2.77–3.25)	1.60 (1.46–1.76)
Breast	0.03 (0.02–0.04)	0.03 (0.03–0.04)
Abdominal	1.23 (1.19–1.26)	0.96 (0.92–1.00)
Urologic	0.37 (0.35–0.39)	0.29 (0.27–0.31)
Gynecologic	0.18 (0.16–0.20)	0.29 (0.26–0.32)
Orthopedic	1.00 (reference)	1.00 (reference)
Vascular	1.24 (1.17–1.31)	0.74 (0.69–0.78)
Dermatologic	1.31 (1.22–1.40)	1.02 (0.94–1.10)

Single and multivariable logistic regressions using ASA

*Abbreviations*: *ASA* American Society of Anesthesiologists, *DM 1* Diabetes mellitus type 1, *DM 2* Diabetes mellitus type 2, *OR* Odds ratio, *CI* Confidence interval

Table 3 shows the same analyses with 1 year mortality as the outcome. The results are similar. Patients with diabetes, and specifically type 1 DM, have an increased risk of death. Higher ASA class, acute surgery, and cancer surgery stand out in their association with mortality at 1 year.

**Table 3:** Crude and adjusted odds ratios for mortality with ASA as a proxy for comorbidity

Characteristics	Crude OR, mortality ± 95% CI	Adjusted OR, mortality ± 95% CI
Non-diabetics	1.00 (reference)	1.00 (reference)
DM, type 1	2.17 (1.96–2.39)	1.74 (1.55–1.93)
DM, type 2	2.27 (2.16–2.39)	1.17 (1.10–1.24)
DM, unspecified	3.07 (1.17–6.74)	1.15 (0.39–2.85)
Age	1.07 (1.07–1.07)	1.05 (1.05–1.05)
Women	0.81 (0.79–0.84)	0.99 (0.95–1.03)
Men	1.00 (reference)	1.00 (reference)
**ASA class**		
ASA 1	1.00 (reference)	1.00 (reference)
ASA 2	5.85 (5.32–6.44)	2.48 (2.25–2.74)
ASA 3	23.60 (21.50–25.90)	6.21 (5.63–6.86)
ASA 4	50.00 (44.90–55.80)	9.92 (8.83–11.2)
Year of surgery		
2007–2010	1.00 (reference)	1.00 (reference)
2011–2014	0.84 (0.82–0.87)	0.74 (0.71–0.76)
Type of surgery		
Acute	2.43 (2.35–2.51)	2.43 (2.33–2.54)
Elective	1.00 (reference)	1.00 (reference)
Cancer	4.20 (4.06–4.35)	6.22 (5.95–6.51)
Neuro	1.47 (1.39–1.56)	1.50 (1.40–1.61)
Endocrine	0.22 (0.18–0.27)	0.57 (0.46–0.70)
Ophthalmic	0.44 (0.35–0.54)	0.82 (0.65–1.02)
Ear, nose, and throat	0.27 (0.22–0.33)	0.63 (0.51–0.76)
Oral and maxillofacial	0.39 (0.34–0.44)	0.80 (0.68–0.93)
Thoracic non-cardiac	2.00 (1.77–2.26)	1.07 (0.93–1.22)
Breast	0.29 (0.25–0.33)	0.23 (0.20–0.27)
Abdominal	1.34 (1.29–1.40)	1.01 (0.96–1.07)
Urologic	1.09 (1.03–1.15)	0.63 (0.59–0.68)
Gynecologic	0.35 (0.32–0.39)	0.53 (0.48–0.60)
Orthopedic	1.00 (reference)	1.00 (reference)
Vascular	1.49 (1.39–1.60)	0.99 (0.91–1.08)
Dermatologic	1.15 (1.04–1.27)	0.74 (0.66–0.83)

Single and multivariable logistic regressions using ASA

*Abbreviations*: *ASA* American Society of Anesthesiologists, *DM 1* Diabetes mellitus type 1, *DM 2* Diabetes mellitus type 2, *OR* Odds ratio, *CI* Confidence interval

Additional file 1: Tables S1 (low DAH30) and S2 (1 year mortality) use ICD codes to adjust for comorbidities. They demonstrate overall similar results, with DM type 1 associated with both DAH30 < 15 and risk of death. Again, acute surgery is a risk factor, as are certain ICD-based comorbid conditions.

In the supplements, two Kaplan–Meier graphs (Additional file 1: Figs. S1 and S2) detail the long-term mortality for patients undergoing surgery with DM type 1 and 2. Both subtypes of diabetes have a continuously increased mortality the year after index surgery.

## Discussion

This large national cohort study of patients undergoing major surgery found that patients with diabetes had significantly lower DAH30 as compared to those without diabetes. Patients with DM were older and had higher comorbid burden and needed more emergency surgery. Even after adjustment for illness severity and age, the odds for having a DAH30 below 15, indicating death and/or complications, was increased for both type 1 and type 2 diabetes. In the year following the index surgery, DM patients had a higher mortality as compared to those with no diabetes.

This study differs from previous efforts to characterize the perioperative outcomes of patients with diabetes, in that we use DAH30, a composite “catch-all” metric (Bell et al. 2019). There is a risk of underestimating the number of postoperative complications—wound infections, myocardial injury, acute kidney injury, and respiratory failure—when using a database with close to 300,000 patients and relying on EMR data. However, in previous studies, we found an incremental increase in 30-day complication rates, and a decrease in 1-year survival, as days at home decreased (Bell et al. 2019). The DM patients in the present study had a mean DAH30 that was around three (days) lower than for non-diabetic patients. As DAH30 both accounts for length of stay and readmissions our findings are—to a degree—in line with previous studies. In a meta-analysis, patients with DM had an OR of 1.99 for extended LOS, when compared to non-diabetics (Zhang et al. 2022). The same study found that readmissions were more common in the DM group, OR 1.40 (Zhang et al. 2022). Similarly, a meta-analysis on colorectal surgery found that DM had an OR of readmission of 1.40 (Tan et al. 2021). Further indicating that our use of DAH30 as a broad measure of perioperative outcomes for patients with diabetes has benefits is this aforementioned scoping review from 2022; it reported how only 5 of 7 studies showed DM to be associated with mortality, 5/13 reported an association with “complications” (as a composite measure), and 12/17 studies found DM was associated with “infection” (Drayton et al. 2022). The same review found large inconsistencies in the definition of diabetes mellitus in the literature (Drayton et al. 2022). A study in China of 1525 patients undergoing orthopedic or general surgery showed that diabetic patients had a higher risk of surgical complications than non-diabetics; this risk was more pronounced if the patient had documented diabetic complications and postoperative high blood glucose (BG) (Wang et al. 2019). In a case–control study of patients undergoing non-cardiac, non-vascular surgery, elevated preoperative glucose levels were associated with increased perioperative mortality. Glucose levels in the diabetes range were associated with doubled all-cause mortality (Noordzij et al. 2007). In the absence of BG, we used both ICD and ATC codes to subgroup patients to type 1 and 2 diabetes. As expected, the type 2 patients were much older, but overall DAH30 was quite similar between the groups.

There are a number of clinical implications of our results. Only measuring complications is unlikely to fully capture the patient experience or potential recovery after surgery, but avoiding extra days in hospital after surgery or acute illness is highly valued by most patients (Hinami et al. 2014; Fried et al. 2002; Xian et al. 2015). There are healthcare and societal effects of longer in-hospital stay and/or readmissions and as previously demonstrated DAH30 correlates with perioperative costs (Reilly et al. 2022). It is possible that preoperative assessment and risk profiling for diabetes and subsequent perioperative optimization for patients with diabetes would improve surgical outcomes.

This study has inherent weaknesses. Data stems from a dataset from 2007 to 2014 with 1 year of follow-up. The improved DAH30 and mortality outcomes in the latter part of the study time frame indicate that treatment for diabetic patients is improving, and even more up-to-date studies are needed. More importantly, we lack BG measurements and glycated hemoglobin A1c (HbA1c) since our dataset was not cross-matched with the Swedish diabetes quality register. All registry-based studies may be affected by data entry errors. There are strengths, primarily based on high-resolution and complete perioperative data across a large number of Swedish hospitals, ranging from smaller regional institutions to major university hospitals. Overall Swedish health care registry coverage uniquely allowed us to characterize preoperative comorbidities, specifically with the combination of ICD and ATC codes to determine if the patients had type 1 or type 2 DM. We could also capture both patient risk factors and surgical risk factors, as well as process of care outcomes and long-term mortality. It is to the best of our knowledge the first time DAH30 has been used as an outcome metric in patients with diabetes. Lastly, the study cohort size is on par with some of the largest retrospective studies in the field, thus permitting inference with high statistical power and adjustment for multiple covariates. Generalizability should be high, in particular in developed countries.

## Conclusions

Diabetes mellitus is common among patients undergoing surgery. The patient-centered outcome metric DAH30 is significantly lowered among both patients with type 1 and type 2 diabetes. Long-term mortality is elevated among these patients.

### Supplementary Information

**Additional file 1: Table S1.** Univariate and multivariable logistic regression for low DAH30 with ICD-10 codes as proxy for comorbidity. **Table S2.** Univariate and multivariable logistic regression for one year mortality with ICD-10 codes as proxy for comorbidity. **Fig. S1.** Kaplan Meier curve for one year mortality for DM 1 vs nondiabetics. **Fig. S2.** Kaplan Meier curve for one year mortality for DM 2 vs nondiabetics.

## Data Availability

The datasets generated and/or analyzed during the current study are not publicly available but are available from the corresponding author upon reasonable request.
