# Supplementary material for: Days at home alive after major surgery in patients with and without diabetes: an observational cohort study
Source: Perioper Med (Lond). 2024 Jan 22;13:4. doi: 10.1186/s13741-023-00357-5 (PMC10802053; doi:10.1186/s13741-023-00357-5)
Supplement: Supplementary file 1 — Additional file 1: Table S1. Univariate and multivariable logistic regression for low DAH30 with ICD-10 codes as proxy for comorbidity. Table S2. Univariate and multivariable logistic regression for one year mortality with ICD-10 codes as proxy for comorbidity. Fig. S1. Kaplan Meier curve for one year mortality for DM 1 vs nondiabetics. Fig. S2. Kaplan Meier curve for one year mortality for DM 2 vs nondiabetics. [file 13741_2023_357_MOESM1_ESM.docx]

### **SUPPLEMENTAL MATERIAL**

**sTable 1. Univariate and multivariable logistic regression for low DAH30 with ICD-10 codes as proxy for comorbidity.**

| Characteristics | Crude OR, DAH30 < 15 ± 95% CI | P value, crude OR, DAH30 < 15 ^§^ | Adjusted OR, DAH30 < 15 ± 95% CI | P value, adjusted OR, DAH30 < 15 ^§^ |
| --- | --- | --- | --- | --- |
| Nondiabetics | 1.00 (reference) | (reference) | 1.00 (reference) | (reference) |
| DM, type 1 | 2.17 (2.01-2.33) | **<0.001**^*^ | 1.63 (1.50-1.78) | **<0.001**^*^ |
| DM, type 2 | 2.14 (2.06-2.23) | **<0.001**^*^ | 1.31 (1.25-1.37) | **<0.001**^*^ |
| DM, unspecified | 3.67 (1.85-6.85) | **<0.001**^*^ | 1.56 (0.72-3.22) | 0.2^*^ |
| Age | 1.04 (1.04-1.04) | **<0.001**^†^ | 1.03 (1.03-1.03) | **<0.001**^†^ |
| Women | 0.80 (0.78-0.82) | **<0.001**^*^ | 0.97 (0.94-1.00) | **0.027**^*^ |
| Men | 1.00 (reference) | (reference) | 1.00 (reference) | (reference) |
| **Preoperative data** |  |  |  |  |
| No history of heart-, arrhythmic-, cardiovascular-, cerebrovascular-, peripheral artery-, renal- or lung disease | 1.00 (reference) | (reference) | 1.00 (reference) | (reference) |
| Heart disease | 2.98 (2.87-3.09) | **<0.001**^*^ | 1.31 (1.25-1.37) | **<0.001**^*^ |
| Arrhythmic disease | 2.56 (2.48-2.63) | **<0.001**^*^ | 1.11 (1.06-1.16) | **<0.001**^*^ |
| Cardiovascular disease | 2.19 (2.13-2.24) | **<0.001**^*^ | 1.11 (1.07-1.14) | **<0.001**^*^ |
| Cerebrovascular disease | 2.75 (2.64-2.87) | **<0.001**^*^ | 1.24 (1.18-1.30) | **<0.001**^*^ |
| Peripheral artery disease | 2.77 (2.65-2.89) | **<0.001**^*^ | 1.76 (1.67-1.85) | **<0.001**^*^ |
| Renal disease | 2.46 (2.36-2.57) | **<0.001**^*^ | 1.65 (1.57-1.73) | **<0.001**^*^ |
| Lung disease | 2.66 (2.56-2.76) | **<0.001**^*^ | 1.29 (1.22-1.36) | **<0.001**^*^ |
| **Year of surgery** |  |  |  |  |
| 2007-2010 | 1.00 (reference) | (reference) | 1.00 (reference) | (reference) |
| 2011-2014 | 0.91 (0.89-0.93) | **<0.001**^*^ | 0.78 (0.76-0.80) | **<0.001**^*^ |
| **Acuity of surgery** |  |  |  |  |
| Acute | 3.25 (3.17-3.33) | **<0.001**^*^ | 3.24 (3.13-3.35) | **<0.001**^*^ |
| Elective | 1.00 (reference) | (reference) | 1.00 (reference) | (reference) |
| **Type of surgery** |  |  |  |  |
| Orthopedic | 1.00 (reference) | (reference) | 1.00 (reference) | (reference) |
| Cancer | 1.61 (1.57-1.65) | **<0.001**^*^ | 3.24 (3.13-3.35) | **<0.001**^*^ |
| Neuro | 3.84 (3.72-3.97) | **<0.001**^*^ | 4.44 (4.26-4.62) | **<0.001**^*^ |
| Endocrine | 0.09 (0.07-0.11) | **<0.001**^*^ | 0.19 (0.15-0.23) | **<0.001**^*^ |
| Ophthalmic | 0.12 (0.09-0.15) | **<0.001**^*^ | 0.13 (0.10-0.18) | **<0.001**^*^ |
| Ear, nose and throat | 0.19 (0.16-0.22) | **<0.001**^*^ | 0.37 (0.31-0.43) | **<0.001**^*^ |
| Oral and maxillofacial | 0.19 (0.17-0.22) | **<0.001**^*^ | 0.35 (0.31-0.40) | **<0.001**^*^ |
| Thoracic non cardiac | 3.00 (2.77-3.25) | **<0.001**^*^ | 2.51 (2.29-2.75) | **<0.001**^*^ |
| Breast | 0.03 (0.02-0.04) | **<0.001**^*^ | 0.03 (0.02-0.04) | **<0.001**^*^ |
| Abdominal | 1.23 (1.19-1.26) | **<0.001**^*^ | 0.99 (0.96-1.03) | 0.7^*^ |
| Urologic | 0.37 (0.35-0.39) | **<0.001**^*^ | 0.26 (0.24-0.28) | **<0.001**^*^ |
| Gynecologic | 0.18 (0.16-0.20) | **<0.001**^*^ | 0.28 (0.25-0.30) | **<0.001**^*^ |
| Vascular | 1.24 (1.17-1.31) | **<0.001**^*^ | 0.77 (0.72-0.82) | **<0.001**^*^ |
| Dermatologic | 1.31 (1.22-1.40) | **<0.001**^*^ | 1.04 (0.97-1.12) | 0.3^*^ |

Univariate and multivariable logistic regressions using ICD-10. Abbreviations: ASA = American society of anesthesiologists, DM = Diabetes mellitus, OR = Odds ratio, CI = Confidence interval, ICD-10 = International classification of disease, tenth edition. ^§^Significant p values bolded. ^*^Pearson´s chi squared test. ^†^Unpaired two sample t-test.

**sTable 2. Univariate and multivariable logistic regression for one year mortality with ICD-10 codes as proxy for comorbidity.**

| Characteristics | Crude OR mortality ± 95% CI | P value, crude OR, mortality ^§^ | Adjusted OR, mortality ± 95% CI | P value, adjusted OR, mortality ^§^ |
| --- | --- | --- | --- | --- |
| *Nondiabetics* | 1.00 (reference) | (reference) | 1.00 (reference) | (reference) |
| *DM, type 1* | 2.17 (1.96-2.39) | **<0.001**^*^ | 1.78 (1.59-1.99) | **<0.001**^*^ |
| *DM, type 2* | 2.27 (2.16-2.39) | **<0.001**^*^ | 1.24 (1.17-1.31) | **<0.001**^*^ |
| *DM, unspecified* | 3.07 (1.17-6.74) | **0.011**^*^ | 1.08 (0.38-2.67) | 0.9^*^ |
| *Age* | 1.07 (1.07-1.07) | **<0.001**^†^ | 1.06 (1.05-1.06) | **<0.001**^†^ |
| *Women* | 0.81 (0.79-0.84) | **<0.001**^*^ | 1.02 (0.98-1.07) | 0.2^*^ |
| *Men* | 1.00 (reference) | (reference) | 1.00 (reference) | (reference) |
| ***Preoperative data*** |  |  |  |  |
| *No history of heart-, arrhythmic-, cardiovascular-, cerebrovascular-, peripheral artery-, renal- or lung disease* | 1.00 (reference) | (reference) | 1.00 (reference) | (reference) |
| *Heart disease* | 3.88 (3.71-4.06) | **<0.001**^*^ | 1.37 (1.30-1.45) | **<0.001**^*^ |
| *Arrhythmic disease* | 3.49 (3.36-3.62) | **<0.001**^*^ | 1.29 (1.22-1.37) | **<0.001**^*^ |
| *Cardiovascular disease* | 2.64 (2.55-2.73) | **<0.001**^*^ | 1.01 (0.97-1.06) | 0.6^*^ |
| *Cerebrovascular disease* | 2.97 (2.82-3.14) | **<0.001**^*^ | 1.32 (1.24-1.40) | **<0.001**^*^ |
| *Peripheral artery disease* | 2.68 (2.53-2.84) | **<0.001**^*^ | 1.44 (1.35-1.54) | **<0.001**^*^ |
| *Renal disease* | 3.30 (3.14-3.48) | **<0.001**^*^ | 1.85 (1.74-1.96) | **<0.001**^*^ |
| *Lung disease* | 3.33 (3.18-3.49) | **<0.001**^*^ | 1.31 (1.22-1.40) | **<0.001**^*^ |
| ***Year of surgery*** |  |  |  |  |
| *2007-2010* | 1.00 (reference) | (reference) | 1.00 (reference) | (reference) |
| *2011-2014* | 0.84 (0.82-0.87) | **<0.001**^*^ | 0.76 (0.73-0.79) | **<0.001**^*^ |
| ***Acuity of surgery*** |  |  |  |  |
| *Acute* | 2.43 (2.35-2.51) | **<0.001**^*^ | 2.84 (2.72-2.96) | **<0.001**^*^ |
| *Elective* | 1.00 (reference) | (reference) | 1.00 (reference) | (reference) |
| ***Type of surgery*** |  |  |  |  |
| *Orthopedic* | 1.00 (reference) | (reference) | 1.00 (reference) | (reference) |
| *Cancer* | 4.20 (4.06-4.35) | **<0.001**^*^ | 7.27 (6.95-7.61) | **<0.001**^*^ |
| *Neuro* | 1.47 (1.39-1.56) | **<0.001**^*^ | 2.12 (1.98-2.27) | **<0.001**^*^ |
| *Endocrine* | 0.22 (0.18-0.27) | **<0.001**^*^ | 0.59 (0.48-0.73) | **<0.001**^*^ |
| *Ophthalmic* | 0.44 (0.35-0.54) | **<0.001**^*^ | 0.77 (0.61-0.96) | **0.026**^*^ |
| *Ear, nose and throat* | 0.27 (0.22-0.33) | **<0.001**^*^ | 0.60 (0.49-0.74) | **<0.001**^*^ |
| *Oral and maxillofacial* | 0.39 (0.34-0.44) | **<0.001**^*^ | 0.78 (0.67-0.91) | **0.001**^*^ |
| *Thoracic non cardiac* | 2.00 (1.77-2.26) | **<0.001**^*^ | 1.41 (1.22-1.61) | **<0.001**^*^ |
| *Breast* | 0.29 (0.25-0.33) | **<0.001**^*^ | 0.19 (0.17-0.22) | **<0.001**^*^ |
| *Abdominal* | 1.34 (1.29-1.40) | **<0.001**^*^ | 1.03 (0.97-1.08) | 0.4^*^ |
| *Urologic* | 1.09 (1.03-1.15) | **0.002**^*^ | 0.55 (0.51-0.59) | **<0.001**^*^ |
| *Gynecologic* | 0.35 (0.32-0.39) | **<0.001**^*^ | 0.51 (0.46-0.57) | **<0.001**^*^ |
| *Vascular* | 1.49 (1.39-1.60) | **<0.001**^*^ | 0.96 (0.88-1.05) | 0.3^*^ |
| *Dermatologic* | 1.15 (1.04-1.27) | **0.004**^*^ | 0.71 (0.63-0.79) | **<0.001**^*^ |

Univariate and multivariable logistic regressions using ICD-10. Abbreviations: ASA = American society of anesthesiologists, DM = Diabetes mellitus, OR = Odds ratio, CI = Confidence interval, ICD-10 = International classification of disease, tenth edition. ^§^Significant p values bolded. ^*^Pearson´s chi squared test. ^†^Unpaired two sample t-test.

**Supplemental figure 1. Kaplan Meier curve for one year mortality for DM 1 vs nondiabetics.**


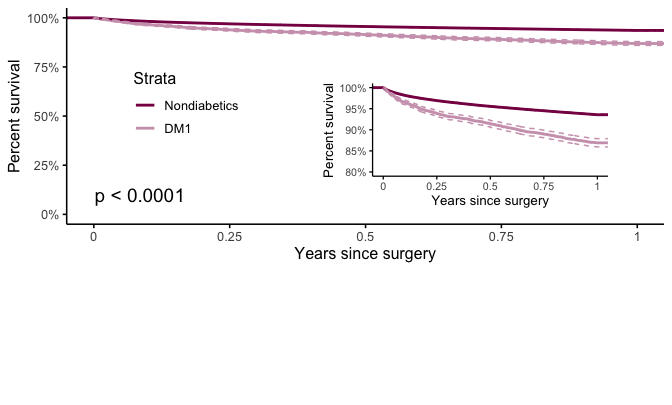


Kaplan Meier curve for DM 1 compared to nondiabetics. The dotted lines represent 95% CI. The smaller plot is zoomed to only show 80-100% survival rates. Abbreviations: CI = Confidence interval, DM 1 = Diabetes mellitus type 1.

**Supplemental figure 2. Kaplan Meier curve for one year mortality for DM 2 vs nondiabetics.**


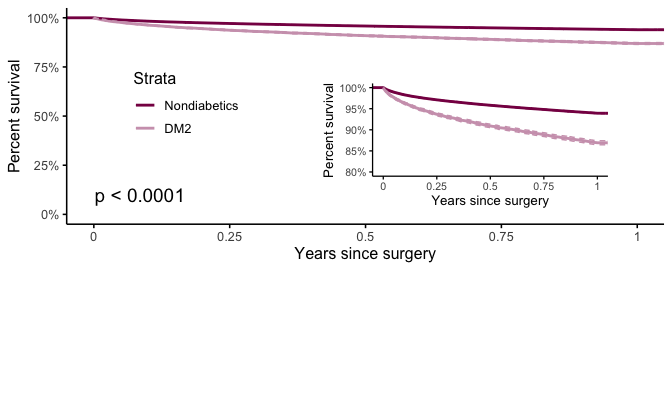


Kaplan Meier curve for DM 2 compared to nondiabetics. The dotted lines represent 95% CI. The smaller plot is zoomed to only show 80-100% survival rates. Abbreviations: CI = Confidence interval, DM 2 = Diabetes mellitus type 2.

**ALTERNATIVE Table 2. Univariate and multivariable logistic regression for low DAH30 with ASA as proxy for comorbidity.**

| Characteristics | Crude OR, DAH30 < 15 ± 95% CI | Adjusted OR, DAH30 < 15 ± 95% CI |
| --- | --- | --- |
| *Nondiabetics* | 1.00 (reference) | 1.00 (reference) |
| *DM, type 1* | 2.17 (2.01-2.33) | 1.42 (1.30-1.54) |
| *DM, type 2* | 2.14 (2.06-2.23) | 1.13 (1.09-1.19) |
| *DM, unspecified* | 3.67 (1.85-6.85) | 1.44 (0.66-3.01) |
| *Age* | 1.04 (1.04-1.04) | 1.01 (1.01-1.02) |
| *Men* | 1.00 (reference) | 1.00 (reference) |
| *Women* | 0.80 (0.78-0.82) | 0.96 (0.94-0.99) |
| ***ASA class*** |  |  |
| *ASA 1* | 1.00 (reference) | 1.00 (reference) |
| *ASA 2* | 3.66 (3.47-3.86) | 2.67 (2.52-2.83) |
| *ASA 3* | 15.20 (14.40-16.00) | 7.32 (6.91-7.76) |
| *ASA 4* | 68.70 (64.00-73.70) | 25.60 (23.70-27.60) |
| ***Year of surgery*** |  |  |
| *2007-2010* | 1.00 (reference) | 1.00 (reference) |
| *2011-2014* | 0.91 (0.89-0.93) | 0.75 (0.73-0.77) |
| ***Acuity of surgery*** |  |  |
| *Elective* | 1.00 (reference) | 1.00 (reference) |
| *Acute* | 3.25 (3.17-3.33) | 2.71 (2.63-2.79) |
| ***Type of surgery*** |  |  |
| *Orthopedic* | 1.00 (reference) | 1.00 (reference) |
| *Cancer* | 1.61 (1.57-1.65) | 2.75 (2.65-2.85) |
| *Neuro* | 3.84 (3.72-3.97) | 2.90 (2.78-3.03) |
| *Endocrine* | 0.09 (0.07-0.11) | 0.17 (0.13-0.20) |
| *Ophthalmic* | 0.12 (0.09-0.15) | 0.14 (0.11-0.19) |
| *Ear, nose and throat* | 0.19 (0.16-0.22) | 0.36 (0.31-0.42) |
| *Oral and maxillofacial* | 0.19 (0.17-0.22) | 0.35 (0.31-0.40) |
| *Thoracic non cardiac* | 3.00 (2.77-3.25) | 1.60 (1.46-1.76) |
| *Breast* | 0.03 (0.02-0.04) | 0.03 (0.03-0.04) |
| *Abdominal* | 1.23 (1.19-1.26) | 0.96 (0.92-1.00) |
| *Urologic* | 0.37 (0.35-0.39) | 0.29 (0.27-0.31) |
| *Gynecologic* | 0.18 (0.16-0.20) | 0.29 (0.26-0.32) |
| *Vascular* | 1.24 (1.17-1.31) | 0.74 (0.69-0.78) |
| *Dermatologic* | 1.31 (1.22-1.40) | 1.02 (0.94-1.10) |

Univariate and multivariable logistic regressions using ASA. Abbreviations: ASA = American society of anesthesiologists, DM 1 = Diabetes mellitus type 1, DM 2 = Diabetes mellitus type 2, OR = Odds ratio, CI = Confidence interval.

**ALTERNATIVE Table 3. Univariate and multivariable logistic regression for one year mortality with ASA as proxy for comorbidity.**

| Characteristics | Crude OR, mortality ± 95% CI | Adjusted OR, mortality ± 95% CI |
| --- | --- | --- |
| *Nondiabetics* | 1.00 (reference) | 1.00 (reference) |
| *DM, type 1* | 2.17 (1.96-2.39) | 1.74 (1.55-1.93) |
| *DM, type 2* | 2.27 (2.16-2.39) | 1.17 (1.10-1.24) |
| *DM, unspecified* | 3.07 (1.17-6.74) | 1.15 (0.39-2.85) |
| *Age* | 1.07 (1.07-1.07) | 1.05 (1.05-1.05 |
| *Men* | 1.00 (reference) | 1.00 (reference) |
| *Women* | 0.81 (0.79-0.84) | 0.99 (0.95-1.03) |
| ***ASA class*** |  |  |
| *ASA 1* | 1.00 (reference) | 1.00 (reference) |
| *ASA 2* | 5.85 (5.32-6.44) | 2.48 (2.25-2.74) |
| *ASA 3* | 23.60 (21.50–25.90) | 6.21 (5.63-6.86) |
| *ASA 4* | 50.00 (44.90–55.80) | 9.92 (8.83-11.2) |
| ***Year of surgery*** |  |  |
| *2007-2010* | 1.00 (reference) | 1.00 (reference) |
| *2011-2014* | 0.84 (0.82-0.87) | 0.74 (0.71-0.76) |
| ***Acuity of surgery*** |  |  |
| *Elective* | 1.00 (reference) | 1.00 (reference) |
| *Acute* | 2.43 (2.35-2.51) | 2.43 (2.33-2.54) |
| ***Type of surgery*** |  |  |
| *Orthopedic* | 1.00 (reference) | 1.00 (reference) |
| *Cancer* | 4.20 (4.06-4.35) | 6.22 (5.95-6.51) |
| *Neuro* | 1.47 (1.39-1.56) | 1.50 (1.40-1.61) |
| *Endocrine* | 0.22 (0.18-0.27) | 0.57 (0.46-0.70) |
| *Ophthalmic* | 0.44 (0.35-0.54) | 0.82 (0.65-1.02) |
| *Ear, nose and throat* | 0.27 (0.22-0.33) | 0.63 (0.51-0.76) |
| *Oral and maxillofacial* | 0.39 (0.34-0.44) | 0.80 (0.68-0.93) |
| *Thoracic non cardiac* | 2.00 (1.77-2.26) | 1.07 (0.93-1.22) |
| *Breast* | 0.29 (0.25-0.33) | 0.23 (0.20-0.27) |
| *Abdominal* | 1.34 (1.29-1.40) | 1.01 (0.96-1.07) |
| *Urologic* | 1.09 (1.03-1.15) | 0.63 (0.59-0.68) |
| *Gynecologic* | 0.35 (0.32-0.39) | 0.53 (0.48-0.60) |
| *Vascular* | 1.49 (1.39-1.60) | 0.99 (0.91-1.08) |
| *Dermatologic* | 1.15 (1.04-1.27) | 0.74 (0.66-0.83) |

Univariate and multivariable logistic regressions using ASA. Abbreviations: ASA = American society of anesthesiologists, DM 1 = Diabetes mellitus type 1, DM 2 = Diabetes mellitus type 2, OR = Odds ratio, CI = Confidence interval.
